# High-frequency oscillatory ventilation and short-term outcome in neonates and infants undergoing cardiac surgery: a propensity score analysis

**DOI:** 10.1186/cc10521

**Published:** 2011-10-28

**Authors:** Mirela Bojan, Simone Gioanni, Philippe Mauriat, Philippe Pouard

**Affiliations:** 1 Anesthesiolgy and Critical Care Department, Necker-Enfants Malades Hospital, Assistance Publique-Hôpitaux de Paris, 149 rue de Sèvres, 75015 Paris, France; 2 Anesthesiolgy and Critical Care Department, Haut-Lévêque Hospital, 1 avenue de Magellan, 33604 Pessac, France

## Abstract

**Introduction:**

Experience with high-frequency oscillatory ventilation (HFOV) after congenital cardiac surgery is limited despite evidence about reduction in pulmonary vascular resistance after the Fontan procedure. HFOV is recommended in adults and children with acute respiratory distress syndrome. The aim of the present study was to assess associations between commencement of HFOV on the day of surgery and length of mechanical ventilation, length of Intensive Care Unit (ICU) stay and mortality in neonates and infants with respiratory distress following cardiac surgery.

**Methods:**

A logistic regression model was used to develop a propensity score, which accounted for the probability of being switched from conventional mechanical ventilation (CMV) to HFOV on the day of surgery. It included baseline characteristics, type of procedure and postoperative variables, and was used to match each patient with HFOV with a control patient, in whom CMV was used exclusively. Length of mechanical ventilation, ICU stay and mortality rates were compared in the matched set.

**Results:**

Overall, 3,549 neonates and infants underwent cardiac surgery from January 2001 through June 2010, 120 patients were switched to HFOV and matched with 120 controls. After adjustment for the delay to sternal closure, duration of renal replacement therapy, occurrence of pulmonary hypertension and year of surgery, the probability of successful weaning over time and the probability of ICU delivery over time were significantly higher in patients with HFOV, adjusted hazard ratios and 95% confidence intervals: 1.63, 1.17 to 2.26 (*P *= 0.004). and 1.65, 95% confidence intervals: 1.20 to 2.28 (*P *= 0.002) respectively. No association was found with mortality.

**Conclusions:**

When commenced on the day of surgery in neonates and infants with respiratory distress following cardiac surgery, HFOV was associated with shorter lengths of mechanical ventilation and ICU stay than CMV.

## Introduction

High-frequency oscillatory ventilation (HFOV) is an established treatment for acute respiratory distress in preterm neonates. However, there is no evidence that it improves outcome in term or near-term neonates with pulmonary disease [1]. HFOV is considered as a rescue therapy in children with severe acute respiratory distress syndrome (ARDS), but to date there is lack of evidence to support it [2,3]. HFOV is also used to achieve lung recruitment and improve oxygenation when recruitment maneuvers have failed, as part of the "open lung" and lung protective ventilation strategies in adults with severe ARDS; early initiation of HFOV has been associated with improved outcome [4-6]. Mild acute lung injury occurs in 12% of adults following cardiopulmonary bypass (CPB), and more severe lung injury, indistinguishable from ARDS, in 0.4% [7,8], as a result of accumulation of excessive extrapulmonary lung water, decreased lung compliance, atelectasis and increased shunting.

Experience with HFOV following cardiac surgery is limited, due to concerns about hemodynamic impairment in animal and human studies [6,9-13]. However, HFOV has been associated with a significant reduction in pulmonary vascular resistance (PVR) after the Fontan procedure in children [14]. Thought to be beneficial for gas exchange and PVR, the present authors have used HFOV in neonates and infants with respiratory distress following cardiac surgery since January 2007. The aim of the present study was to assess associations between commencement of HFOV on the day of surgery (Day 0) and the length of mechanical ventilation and Intensive Care Unit (ICU) stay, and mortality in this population.

## Materials and methods

This retrospective cohort study was conducted at the Necker University Hospital in Paris, France. It was reviewed and approved by the Ethics Committee of the French Society of Thoracic and Cardiovascular Surgery, which waived the requirement for consent to use anonymized records. All parents had provided informed consent to surgery.

Records of all neonates and infants who underwent cardiac surgery between 1 January 2001 and 30 June 2010 were reviewed; patients switched to HFOV on Day 0 were identified as the HFOV group. Those switched to HFOV after Day 0, as a rescue therapy, were not analyzed. The remaining patients were included in the control group. Data for each patient were extracted retrospectively from a prospective database, which is updated daily by the clinical staff. These concerned: demographics, surgical and CPB techniques, short-term outcome variables accounting for the severity of the postoperative illness, such as re-operation, delayed sternal closure, extracorporeal membrane oxygenation (ECMO), acute kidney injury requiring renal replacement therapy (RRT), and hospital-acquired pneumonia [15], length of mechanical ventilation, length of ICU stay and in-hospital mortality. Normothermic CPB with intermittent warm blood cardioplegia was performed in every patient during the study period, except in cases where deep hypothermic circulatory arrest (DHCA) was indicated [16]. Pulmonary arterial pressure was measured in every patient, either continuously by a catheter inserted into the pulmonary artery by the end of surgery, or by serial echocardiography. Persistent pulmonary hypertension was noted whenever it occurred during the postoperative course, and inhaled nitric oxide was administered [17].

All patients were initially commenced on pressure controlled conventional mechanical ventilation (CMV) using a SERVO-300 (Siemens-Elema AB, Sweden) before 2002, then a SERVO-i ventilator (Maquet GmbH&Co.KG, Rastatt, Germany). This was set to provide a positive end expiratory pressure of 2 cmH_2_O, a tidal volume of 6 to 8 ml Kg^-1^, and a fraction of inspired oxygen, which was dependent upon the underlying cardiac disease. In the event of severe respiratory failure, recruitment maneuvers by stepwise increase in the mean airway pressure were applied. The patients were switched to HFOV when hypoxemia and acidosis occurred despite increasing alveolar ventilation on CMV, when the tidal volume exceeded 10 ml kg^-1^, or when there was evidence of pulmonary hypertension and right ventricular failure. The decision to switch was made by the attending intensivist. A SLE 2000 or a SLE 5000 HFO ventilator (SLE Ltd, South Croydon, UK) was used. This was set to a mean airway pressure (Paw) of 12 cmH_2_O, an inspiratory to expiratory ratio of 33%, and an oscillation frequency of 8 Hz. Amplitude was adapted to achieve adequate chest wall vibrations. All parameters were adjusted to achieve optimal inflation, a PaCO2 of 35 to 45 mmHg and a pH > 7.35. The adequacy of the PaO2 level was judged according to the underlying cardiac disease. Patients were switched back to CMV when these conditions had been achieved with an oscillation frequency ≥10 Hz and a mean Paw ≤10 cmH_2_O. Sedation was achieved through a continuous infusion of midazolam and morphine. Whenever possible, muscular relaxants were avoided and spontaneous breathing was maintained. Catecholamine support (milrinone and epinephrine), fluid support and diuretics were administered as appropriate to achieve hemodynamic stability and a negative fluid balance. All patients were weaned from mechanical ventilation when the underlying indication had resolved and following a successful one-hour trial of spontaneous breathing with a continuous positive pressure of 2 cmH_2_O and a pressure support of 10 cmH_2_O.

### Statistical analysis

After testing for normality, baseline characteristics of the two groups were compared using Student's *t *or Mann-Whitney tests for continuous variables and χ2 or Fisher's exact tests for categorical variables.

The hypothesis tested was that patients switched to HFOV had shorter length of mechanical ventilation and ICU stay and lower mortality rates. To control for the bias due to selection of patients switched to HFOV, 1:1 propensity score matching was carried out [18]. Logistic regression was used to develop a propensity score quantifying the probability for each patient undergoing surgery since January 2007 to be switched to HFOV. This included all baseline and post-operative variables accounting for severity of illness found to be different between groups in univariate analysis (*P *< 0.10), and the "HFOV index". Given the large number of surgical procedures and the absence of guidelines for HFOV in this context, an empirical "HFOV index" was attributed to each procedure. This accounted for the influence of each specific procedure being performed on the probability to be switched to HFOV afterwards, and was calculated as the prevalence of HFOV per procedure between January 2007 and June 2010.

In accordance with previous authors, length of mechanical ventilation and length of ICU stay were modeled as censored variables in survivors, with weaning from mechanical ventilation and ICU delivery as censoring events [19]. The probability of successful weaning from mechanical ventilation over time and the probability of ICU delivery over time were calculated for each group using the Kaplan-Meier method, and compared using the log-rank test. Results were confirmed using a multivariable Cox proportional-hazards model, controlling for variables related to length of ICU stay following pediatric cardiac surgery in a previous study [20], variables unbalanced after matching (*P *< 0.10), for the propensity score and the year of surgery. Adjusted Hazard ratios (HR) with 95% confidence intervals were estimated.

The R statistical package, "Design" and "optmatch" libraries [21] were used for the analyses.

## Results

**Figure 1:**
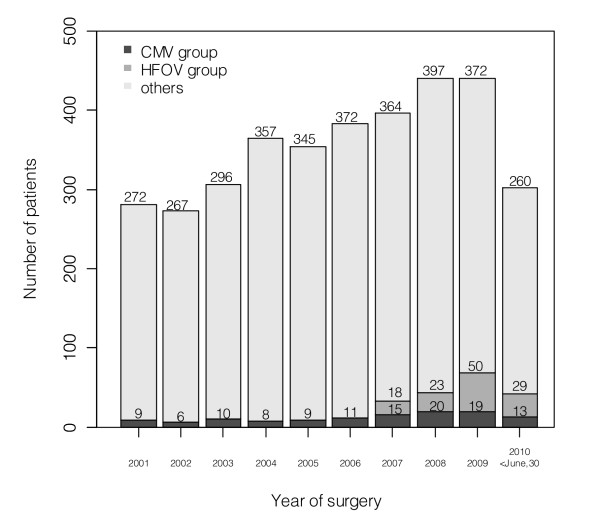
**Number of neonates and infants who underwent surgery during the study period**. The number of patients included in each group after matching is shown on the bottom of each column. High frequency oscillation was used since 2007. CMV, conventional mechanical ventilation; HFOV, high-frequency oscillatory ventilation.

**Figure 2:**
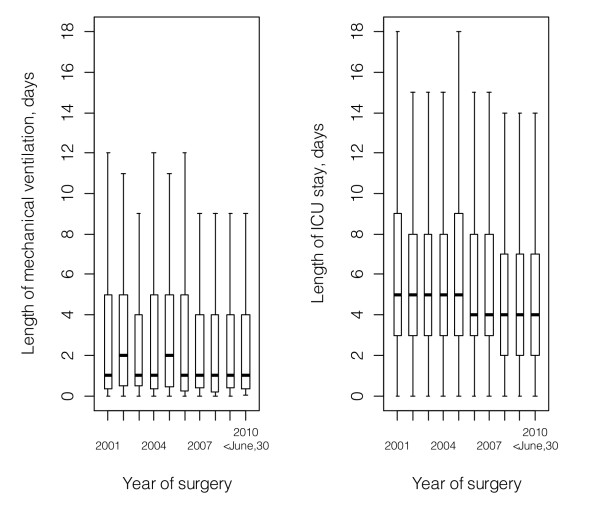
**Length of mechanical ventilation and Intensive Care Unit stay across the study period**. The median values and the inter-quartile ranges were used to construct the boxes. 10^th ^and 90^th ^percentiles are given as whiskers. Outliers are not shown. ICU, Intensive Care Unit.

Overall, 3,549 neonates and infants underwent cardiac surgery during the study period. Life support was withdrawn from four patients with obstructed total anomalous pulmonary venous connection and one patient with severe pulmonary hypoplasia, with hopeless prognosis secondary to pulmonary lymphangienctasia. Another two patients died periprocedural, leaving 3,542 cases to be analyzed. The number of patients, their length of mechanical ventilation and ICU stay across the study period are shown in Figures 1 and 2.

Patient characteristics are shown in Table 1. The 120 neonates and infants switched to HFOV on Day 0 were younger and smaller, had undergone more complex surgery and had experienced more severe postoperative illness. Patients switched to HFOV had longer durations of both mechanical ventilation, median 7 days, inter-quartile ranges (IQR) 5 to 11 vs. 1 day, IQR 0.3 to 4 in controls (*P *< 0.001), and ICU stay, median 11 days, IQR 7 to 15.7 vs. 4 days, IQR 3 to 7 (*P *< 0.001). The median duration of HFOV was 4 days, IQR 2 to 7. The in-hospital mortality rates for the two groups were similar, 8.3% in patients switched to HFOV vs. 4.8% in controls (*P *= 0.08).

**Table 1:** Perioperative patient characteristics before and after matching

	Before matching			After matching	
	HFOV group (*n *= 120)	Overall controls (*n *= 3,422)	*P-*value^a^	CMV group (*n *= 120)	*P-*value^b^
Age (days)	27, 7.7 to 100.2	58, 10 to 149	0.001	33.0, 7.0 to 89.5	0.83
Weight (kg)	3.4, 2.9 to 4.3	3.9, 3.2 to 5.4	< 0.001	3.3, 2.8 to 4.2	0.93
Surgery with cardiopulmonary bypass, n (%)	109 (90.8)	2560 (74.8)	< 0.001	110 (91.7)	0.80
Duration of cardiopulmonary bypass (min)	128.0, 99.5 to 177.0	109.0, 77.0 to 134.0	< 0.001	128.0, 90.0 to 165.0	0.64
Conventional ultrafiltration rate (mL kg^-1 ^h^-1^)	93.3, 69.9 to 120.6	96.6, 66.9, 132.3	0.27	98.3, 87.2 to 127.3	0.40
Aristotle score^c^	9.0, 7.5 to 10.8	8, 6 to 10	< 0.001	9.0, 7.3 to 10.8	0.99
Surgery with deep hypothermic circulatory arrest, n (%)	19 (15.8)	251 (7.3)	< 001	21 (17.5)	0.72
Re-sternotomy, n (%)	19 (15.8)	406 (11.9)	0.19	16 (13.3)	0.59
Requiring re-operation, n (%)	13 (10.8)	177 (5.2)	0.007	16 (13.3)	0.56
Re-operated within 48 hours, n (%)	3 (2.5)	18 (0.5)	0.03	2 (1.7)	0.66
Extracorporeal membrane oxygenation, n (%)	0	23 (0.7)		0	
Requirement for delayed sternal closure, n (%)	56 (46.7)	331 (9.7)	< 0.001	57 (47.5)	0.85
Delay to sternal closure (days)	3, 2 to 4.2	4, 2 to 6	0.21	4, 3 to 7	0.08
Acute kidney injury requiring renal replacement therapy, n (%)	42 (35.0)	127 (3.7)	< 0.001	39 (32.5)	0.58
Requirement for renal replacement therapy on the day of surgery, n (%)	37 (30.8)	89 (2.6)	< 0.001	32 (26.7)	0.36
Duration of renal replacement therapy (days)	2, 1 to 4	3, 2 to 6	0.02	3, 2 to 7	0.09
The propensity score	0.07, 0.02 to 0.31	0.02, 0.01 to 0.03		0.07, 0.02 to 031	

The "HFOV group" included all patients switched to high frequency oscillation on the day of surgery, "Overall controls" included all patients ventilated exclusively conventionally during the study period, and the "CMV group" included the patients ventilated exclusively conventionally in the matched set.

CMV, conventional mechanical ventilation; HFOV, high-frequency oscillatory ventilation.

^a^calculated before matching, using unpaired tests which compared the HFOV group with overall controls

^b^calculated after matching, using paired tests which compared the HFOV group with the CMV group

^c^accounting for the surgical complexity

Data are shown as medians and inter-quartile ranges, or as numbers and percentages.

Table 2 shows the most prevalent procedures and their "HFOV indexes", range 0 to 0.82, median 0.04, IQR 0.03 to 0.08. Table 3 shows the variables included in the propensity score. When data were missing, the median of the respective variable was used (< 5% of all information concerning CPB technique was missing). The propensity score model was well calibrated (Hosmer Lemeshow test, *P *= 0.14) and discriminated well between patients on HFOV and the others (*c *index = 0.82). Patients were more likely to be switched to HFOV on Day 0 if they were small, had undergone a procedure with high "HFOV index", required CPB and DHCA, had hemodynamic impairment precluding closure of the sternum or required RRT on Day 0. Patients commenced on postoperative ECMO were not matched due to their high mortality rate (39.1%).

**Table 2:** Most prevalent procedures in the matched set, along with their "HFOV indexes"

Most prevalent procedures	HFOV group (*n *= 120)	CMV group (*n *= 120)	"HFOV index"^a^
Obstructed TAPVC repair	14	12	0.82
Unrestrictive VSD repair	10	10	0.06
Complete common atrioventricular canal	9	7	0.11
Aortic arch repair	8	7	0.19
Arterial switch operation, VSD repair	6	9	0.08
Truncus arteriosus repair	6	7	0.30
Arterial switch operation	5	8	0.03
Norwood operation	6	6	0.41
Modified Blalock Taussig shunt	5	5	0.09
Tetralogy of Fallot repair	6	4	0.04
Coarctation repair	7	2	0.04
Pulmonary atresia, VSD repair	5	3	0.18
Arterial switch operation, VSD, coarctation repair	3	4	0.18
Bidirectional Glenn	2	4	0.05
Konno Ross procedure	2	4	0.50
Aortic valvuloplasty	3	3	0.10
Other	22	25	

CMV, conventional mechanical ventilation; HFOV, high-frequency oscillatory ventilation; TAPVC, total anomalous pulmonary venous connection; VSD, ventricular septal defect.

^a^accounting for the prevalence of HFOV from 1 January 2007 through 30 June 2010

**Table 3:** Estimates and standard errors for variables included in the propensity score model

Variable	Coefficient estimate	Standard error	*P-v*alue
Intercept	-2.87	0.58	< 0.001
The "HFOV index"^a^	3.94	0.48	< 0.001
Age (days)	0.002	0.002	0.19
Weight (kg)	-0.37	0.12	0.002
Aristotle score^b^	-0.09	0.06	0.10
Surgery with cardiopulmonary bypass	0.87	0.37	0.02
Surgery with deep hypothermic circulatory arrest	-0.97	0.33	0.004
Re-operation	0.59	0.34	0.09
Requirement for a delayed sternal closure	0.81	0.29	0.005
Acute kidney injury requiring renal replacement therapy	0.36	0.62	0.56
Requirement for renal replacement therapy on the day of surgery	1.82	0.63	0.004

The propensity score model included only patients operated from 1 January 2007 through 30 June 2010.

HFOV, high-frequency oscillatory ventilation

^a^calculated as the prevalence of HFOV from 1 January 2007 through 30 June 2010

^b^accounting for the surgical complexity

Matching resulted in two well-balanced groups of 120 patients respectively: the HFOV and the CMV groups (Table 1). The HFOV group had shorter durations of mechanical ventilation, 7 days, IQR 5 to 11 vs 9 days, IQR 5 to 17 in the CMV group (*P *= 0.03), shorter durations of ICU stay, 11 days, IQR 5 to 17 vs 14 days, IQR 9 to 22 (*P *= 0.009), a higher prevalence of pulmonary hypertension, 36.7%, compared to 23.3% (*P *= 0.03) and a similar prevalence of hospital-acquired pneumonia, 49.2% in the HFOV group compared to 47.5% in the CMV group (*P *= 0.80). Ten patients in the HFOV group (8.3%) died during ICU stay, compared to 18 in the CMV group (15.8%) (*P *= 0.08). Median follow-up was 411 days, IQR 32 to 3,060, 18 patients (15.8%) died during follow-up in the HFOV group, compared to 22 in the CMV group (18.3%) (*P *= 0.66).

**Figure 3:**
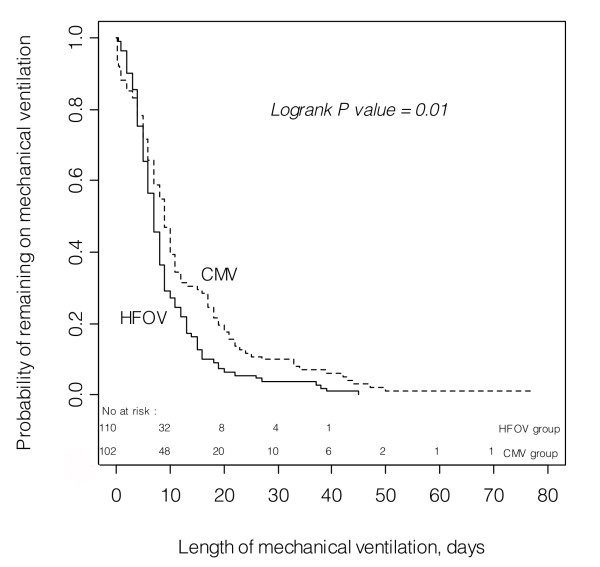
**Kaplan-Meier plots of the probability of successful weaning over time for each ventilation group**. The median length of mechanical ventilation was 7 days in the high-frequency oscillatory group, inter-quartile range 5 to 11, and 9 days in the conventional mechanical ventilation group, inter-quartile range 5 to 17, logrank test = 6.18, *P *= 0.01. CMV, conventional mechanical ventilation; HFOV, high-frequency oscillatory ventilation.

**Figure 4:**
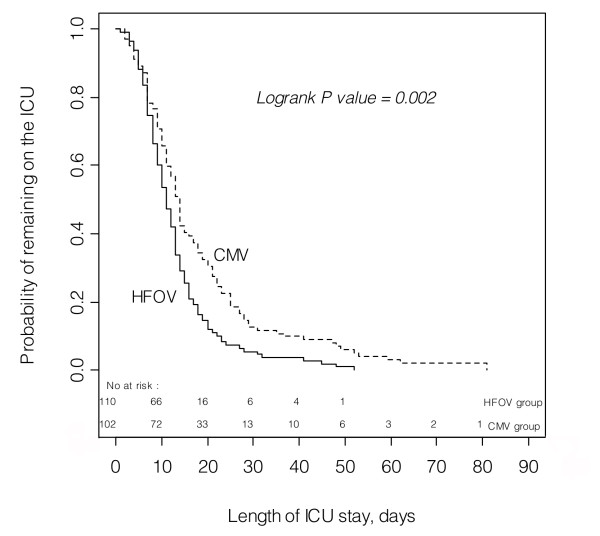
**Kaplan-Meier plots of the probability of ICU delivery over time for each ventilation group**. The median length of ICU stay was 11 days in the high-frequency oscillatory ventilation group, inter-quartile range 7.2 to 15.7 compared with 14 days in the conventional mechanical ventilation group, inter-quartile range 9 to 22, logrank test, 9.39, *P *= 0.002. CMV, conventional mechanical ventilation; HFOV, high-frequency oscillatory ventilation.

Kaplan-Meier plots of the probability of successful weaning from mechanical ventilation over time are shown in Figure 3. The median length of mechanical ventilation was 7 days in the HFOV group, IQR 5 to 11, and 9 days in the CMV group, IQR 5 to 17 (*P *= 0.01). Four patients in the HFOV group underwent mechanical ventilation for ≥ 30 days. Of these, one developed tracheal stenosis and underwent slide tracheoplasty, another required tracheostomy. Ten patients in the CMV group underwent mechanical ventilation for ≥ 30 days. Of these, two developed tracheal stenosis, one of whom died, one developed oeso-tracheal fistula and died, and seven developed chronic lung disease, of whom four required tracheostomy and two died. Kaplan-Meier plots of the probability of ICU delivery over time are shown in Figure 4. The median length of ICU stay was 11 days in the HFOV group, IQR 7.2 to 15.7 and 14 days in the CMV group, IQR 9 to 22 (*P *= 0.002). Differences between length of ventilation and ICU stay were found significant with a statistical power of 0.77 and 0.89, respectively.

Cox proportional-hazards regression analysis, adjusted for the delay to sternal closure, duration of RRT, occurrence of pulmonary hypertension and year of surgery, showed that patients in the HFOV group had a higher probability of successful weaning over time, adjusted HR 1.63; 95% confidence interval (CI) 1.17 to 2.23 (*P *= 0.004) (Table 4). The probability of ICU delivery over time was also higher in the HFOV group, adjusted HR 1.65, 95% CI 1.20 to 2.28 (*P *= 0.002) (Table 4). Longer delay to sternal closure was independently associated with longer length of mechanical ventilation and ICU stay.

**Table 4:** Independent predictors of successful weaning from mechanical ventilation and ICU delivery over time

	Successful weaning from mechanical ventilation			ICU delivery		
Variable	Adjusted Hazard Ratio	95% CI	*P*-value	Adjusted Hazard Ratio	95% CI	*P*-value
HFOV	1.62	1.17 to 2.25	0.004	1.65	1.19 to 2.28	0.002
Delay to sternal closure (days)	0.87	0.82 to 0.93	< 0.001	0.88	0.82 to 0.94	< 0.001
Pulmonary hypertension	0.74	0.54 to 1.02	0.07	0.73	0.53 to 1.01	0.05
Duration of renal replacement therapy (days)	0.95	0.89 to 1.02	0.19	0.94	0.87 to 1.01	0.08
Year of surgery	0.93	0.87 to 0.99	0.03	0.95	0.88 to 1.02	0.16
The propensity score	2.59	1.10 to 6.08	0.03	2.38	0.99 to 5.75	0.05

Adjusted Hazard ratios and 95% CI were estimated using Cox proportional-hazards regression analysis

CI, confidence interval, HFOV, high-frequency oscillatory ventilation

## Discussion

The present study reports experience with HFOV in a population of neonates and infants with respiratory distress following several cardiac surgery procedures. Previous findings reported from randomized trials of HFOV in term or near-term neonates with pulmonary disease showed no benefit in terms of 28-day mortality [1], and our findings were similar. But, unlike previous research on elective use of HFOV, length of mechanical ventilation and length of stay were reduced among patients with a similar severity of illness when they were switched to HFOV on the day of surgery.

### HFOV and PVR

The most common reasons for late weaning from mechanical ventilation following congenital cardiac surgery are a low cardiac output state or a respiratory complication. Even when ventricular function is well preserved and no residual anatomical lesion is present, a low cardiac output may result from inadequate pulmonary blood flow, secondary to elevated PVR. Maintenance of cardiac output by fluid challenge, to ensure adequate preload, leads to extravascular fluid accumulation, pleural and pericardial effusions, pulmonary interstitial edema and decreased compliance. The loss of intravascular volume must be replaced to maintain cardiac output, which may initiate a vicious cycle, and should, therefore, be avoided.

PVR is multifactorial after CPB [22,23] and highly sensitive to changes in intra-thoracic pressure [24] and acidosis [25]. Changes in intra-thoracic pressure have been extensively investigated in the Fontan procedure, where high-frequency ventilation has been found to be associated with an increase of up to 25% in cardiac output and led to halve PVR and mean Paw [14]. Although HFOV is known to be effective in settings leading to hypoxemia, the use has been described in reports of asthma and severe bronchiolitis to treat respiratory acidosis [26,27]. According to Babik *et al*. [23], CPB is responsible for an obstructive process in the bronchi, leading to bronchospasm and acidosis. Bronchospasm is also a frequent postoperative finding in patients with a large preoperative left to right shunt [28]. Thus, the use of HFOV to treat respiratory acidosis in an attempt to decrease PVR after CPB appears justified. In the present study, when switching to HFOV, ventilation frequency was initially set to 8 Hz to promote decarboxylation and, thus, rapidly increase pH. But the retrospective design of the present study rendered collection of reliable data concerning PVR, Paw and gas exchanges impossible. Nevertheless, documented pulmonary hypertension was more prevalent in the HFOV group (before or after transition to HFOV) even after propensity score matching, showing that the HFOV group was still more severely ill. Therefore, the shorter durations of mechanical ventilation in the HFOV group suggested a beneficial effect of HFOV on PVR.

### Hemodynamic status

Usually, HFOV involves slightly higher mean Paw values than CMV, and low cardiac output may occur due to increased pleural pressure and reduced venous return. Studies of HFOV in animal models of ARDS have reported hemodynamic impairment when high airway pressures were applied [9-11]. Studies of adults [6,12,13] and infants [29,30] switched from CMV to HFOV have found effects such as increased pulmonary artery occlusion pressure, increased central venous pressure, and small decreases in cardiac output and stroke volume index, although it was unclear whether these changes were clinically relevant.

By contrast, sedation may lead to excessive venous vasodilatation and impaired venous return following cardiac surgery, whereas spontaneous breathing maintains a negative pleural pressure, facilitates venous return and improves cardiac output. Spontaneous ventilation can be maintained easily in neonates and small children on HFOV without increasing the work of breathing [31,32], thus allowing reduced sedation.

Reliable evaluation of hemodynamic consequences when changing ventilatory settings is impossible in a retrospective study. The low mean Paw HFOV strategy employed in the present study may have allowed the preservation of hemodynamic stability in these patients. Furthermore, if a long delay to sternal closure and a long duration of RRT were considered markers of hemodynamic impairment, then switching to HFOV may have resulted in hemodynamic improvement in the present cohort, since both the delay to sternal closure and the duration of RRT were slightly reduced in the HFOV group (Table 1).

### Limitations

The present study was retrospective and, thus, the validity of the results must be viewed with caution. Attempts were made to minimize bias related to selection of patients switched to HFOV through propensity score matching. Even though, and despite adjustment for the year of surgery, the choice of historical controls cannot rule out bias related to improvements in surgical and medical management of congenital heart diseases throughout the study period. Besides, the choice of transition to HFOV was made by the attending intensivist, and, despite the propensity score methodology employed, we cannot rule out residual bias related to pre-held beliefs about HFOV's performance. Furthermore, analysis of ventilation parameters and hemodynamic consequences were lacking. Because of the various intra-cardiac shunting patterns in the study population, oxygenation indexes were not analyzed. Future studies should address these limitations.

## Conclusions

When commenced on the day of surgery, HFOV was associated with a shorter duration of mechanical ventilation and ICU stay in this population of neonates and infants with respiratory distress following congenital cardiac surgery. No association was observed between the use of HFOV and mortality.

## Key messages

• HFOV has been shown to be associated with lower pulmonary vascular resistance after the Fontan procedure.

• The present study found the use of HFOV to be associated with shorter length of mechanical ventilation and ICU stay in neonates and infants with respiratory distress following several cardiac procedures.

• Since our pathophysiological inferences are drawn from observational results, the beneficial effect of HFOV needs to be confirmed by interventional studies.

## Abbreviations

ARDS: acute respiratory distress syndrome; CI: confidence intervals; CMV: conventional mechanical ventilation; CPB: cardiopulmonary bypass; Day 0: day of surgery; DHCA: deep hypothermic circulatory arrest; IQR: inter-quartile ranges; ECMO: extracorporeal membrane oxygenation; HFOV: high-frequency oscillatory ventilation; HR: hazard ratio; ICU: intensive care unit; Paw: airway pressure; PVR: pulmonary vascular resistance; RRT: renal replacement therapy; TAPVC: total anomalous pulmonary venous connection; VSD: ventricular septal defect.

## Competing interests

The authors declare that they have no competing interests.

## Authors' contributions

MB contributed to the scope and design of the study, obtaining permission for data use, analyzing and interpreting the data, and drafting and revising the manuscript. SG contributed to the scope of the study, to the research concept, and to revising the manuscript. PM and PP contributed both to the scope of the study and to revising the manuscript. All authors have read and approved the manuscript for publication.
